# Differential effect of pulsatile pressure on the myogenic responses of small arteries

**DOI:** 10.1007/s00424-025-03145-w

**Published:** 2025-12-10

**Authors:** André Budrowitz, Mitko Mladenov, Hristo Gagov, Rudolf Schubert

**Affiliations:** 1 Physiology, Institute of Theoretical Medicine, Faculty of Medicine, University of Augsburg, Universitätsstrasse 2, 86159 Augsburg, Germany; 2 Institute of Biology, Faculty of Natural Sciences and Mathematics, University of Ss. Cyril and Methodius, 1000 Skopje, North Macedonia; 3 Department of Animal and Human Physiology, Faculty of Biology, Sofia University ‘St. Kliment Ohridski’, 1164 Sofia, Bulgaria

**Keywords:** Small arteries, Pulsatile pressure, Myogenic response, Calcium sensitivity

## Abstract

The myogenic response is an important regulatory mechanism under physiological as well as pathophysiological conditions. However, little is known about the myogenic response under pulsatile pressure conditions. Therefore, based on the known mechanisms governing the myogenic response induced by static pressure, we tested the hypothesis that a stronger myogenic response induced by pulsatile pressure is due to a larger increase of the intracellular calcium concentration and/or a higher calcium sensitivity of vessel tone. Rat small tail and gracilis arteries were studied using isobaric myography and FURA-2 fluorimetry. We found that in small tail arteries, the effect of pulsatile pressure on the myogenic response is determined by its systolic pressure, whereas in gracilis arteries, the effect of pulsatile pressure is determined by its mean pressure. Interestingly, the effect of pulsatile pressure on the intracellular calcium concentration in both vessels is determined by its systolic pressure. However, while calcium sensitivity of myogenic tone did not differ between static and pulsatile pressure conditions in small tail arteries, it was weaker under pulsatile pressure than under static pressure in gracilis arteries. In conclusion, a stronger myogenic response under pulsatile pressure conditions, i.e., the capability of a vessel to respond to systolic pressure, requires the vessel’s ability to maintain and not lose the calcium sensitivity of myogenic tone compared to static pressure conditions.

## Introduction

The myogenic response of small arteries and arterioles is characterized by vessel constriction in response to an increase in transmural pressure and vessel dilation in response to a decrease in transmural pressure in the absence of any vasoactive, in particular vasoconstrictor substances. The myogenic response contributes to matching the blood perfusion of an organ with its metabolic demand, independent of variations in systemic blood pressure. The myogenic response thus participates in the regulation of blood flow, capillary hydrostatic pressure, and wall tension in many organs in response to changes in perfusion pressure. Furthermore, the myogenic response is altered in several widespread pathological conditions such as chronic heart failure, diabetes, hypertension, and stroke (for reviews see [1–4]). The myogenic response is therefore an important regulatory mechanism under physiological as well as pathophysiological conditions.

Two major, interacting pathways govern the myogenic response. The first pathway is a calcium-dependent pathway based on membrane potential depolarization and a subsequent increase of the intracellular calcium concentration, which is mainly due to calcium influx. The second pathway consists of several signaling mechanisms that regulate the calcium-sensitivity of vessel tone, including PKC and RhoA/Rho kinase/ROS. Both pathways result in an increase of MLC phosphorylation and subsequent vasoconstriction [1–6]. In almost all studies exploring the myogenic response, pressure was changed in a stepwise manner from an initial static to a new static pressure level, which was then maintained for several minutes. Physiologically, however, blood pressure varies in a pulsatile manner. However, little is known about the myogenic response under pulsatile pressure conditions.

Only a few studies have examined the myogenic response evoked by pulsatile pressure. A first study reported that pulse pressure induces a dilation of pig coronary arterioles when the static pressure of 60 mmHg was changed to a pulsatile pressure with a mean value of 60 mmHg and an amplitude of 100 mmHg. This response was suggested to compensate for the compressive effects of heart contraction on coronary arterioles [7]. Later, it was shown that pulsatile pressure induces constriction of rat afferent arterioles when the static pressure of 100 mmHg was changed to a pulsatile pressure with a mean value of 100 mmHg and an amplitude of 40 and 80 mmHg. Extensive additional experiments led to the conclusion that in this vessel the systolic pressure level of the pressure pulse determines the myogenic response under pulsatile pressure conditions. It was proposed that this response protects the capillaries of the glomerulus from potentially damaging effects of systolic pressure, demonstrating the priority of renal protection over renal flow autoregulation [8, 9]. A third type of response to pulsatile pressure was reported in mouse middle cerebral arteries. When static pressure at several pressure levels was changed to pulsatile pressure with the same mean values and an amplitude of 40 mmHg, vessel diameter did not change. It was suggested that this response protects the brain from pressure-induced microvascular injury [10]. The cited data indicate that pulsatile pressure may have different, organ-dependent effects that are distinct from those of static pressure. Unfortunately, the mechanisms underlying this difference in the response of small arterioles to pulsatile pressure are unknown.

Therefore, based on the known mechanisms governing the myogenic response induced by static pressure, we tested the hypothesis that a stronger myogenic response induced by pulsatile pressure is due to a larger increase of the intracellular calcium concentration and/or a higher calcium sensitivity of vessel tone.

## Materials and methods

### Animals

This study was performed on male Wistar rats (16–25 weeks; Charles River Laboratories, Sulzfeld, Germany). The animals were kept at constant 22 °C room temperature and a regulated 12-hour light-dark cycle with free access to standard pellet chow and drinking water. The investigation is in line with the US Guide for the Care and Use of Laboratory Animals (Eighth Edition, National Academy of Sciences, 2011). Approval for the use of laboratory animals in this study was granted by a governmental committee on animal welfare (I-17/17).

### Vessel preparation

The animals were killed under CO_2_ narcosis by decapitation. The small tail artery (running parallel to the commonly examined main tail artery in the depth of the central vascular canal of the tail, for details see Anschütz [11]) and the gracilis artery were isolated in a 4 °C cold physiological salt solution containing (in mM): 145 NaCl, 4.5 KCl, 1.2 NaH_2_PO_4_, 1 MgSO_4_, 0.1 CaCl_2_, 0.025 EDTA and 5 HEPES.

### Isobaric wire myography

Vessels were mounted in a chamber containing the experimental solution (PSS) consisting of (in mmol/L): 120 NaCl, 26 NaHCO_3_, 5.5 glucose, 4.5 KCl, 1.6 CaCl_2_, 1.2 NaH_2_PO_4_, 1.0 MgSO_4_, 0.025 EDTA, and 5 HEPES at pH 7.4 and two mounting pipettes with a tip diameter of about 180 μm. After mounting the vessels, the experimental chamber was placed on the stage of an inverted microscope (Carl Zeiss, Jena, Germany) and continuously perfused by the experimental solution at a rate of 2 ml/min. The microscope image of the vessel was viewed with a CCD camera, digitized by a frame-grabber board, and the inner diameter monitored continuously as described previously [12, 13] at a rate of 10 Hz. With this system, a measurement accuracy of 2 μm per screen pixel was achieved using a 10x objective and a 3.2x projective lens. The pulsatile pressure was produced by a valve connected to the mounting pipettes via a tubing system. This valve managed two fluid reservoirs that were set at the desired diastolic and systolic pressure of the pulsatile pressure to be tested. The valve switched between the two reservoirs at a frequency of 1 Hz. This frequency was selected based on (1) the few previously published reports on the effect of pulsatile pressure on small arteries and (2) technical limitations of the system. The timing of the switching and the elastic properties of the tubing resulted in a pulse pressure with a faster rising and a slower decay phase (this is reflected in the sketch of the pulse pressure waveform shown in the figures). The pulse pressure was characterized by nearly linear rise and decay phases and a rounded transition from the rise to the decay and from the decay to the rise phases. In pilot experiments it was determined that the mean pressure of this pulse pressure could be calculated by: diastolic pressure + ½ (systolic pressure – diastolic pressure). During the experiments, pulse pressure was continuously monitored on an oscilloscope. In case of distortions of the pulse pressure, the corresponding data was discarded.

All experiments were performed without flow through the lumen of the vessels. To ensure this, a horizontally mounted pipette with a small air bubble was inserted into the tubing system. Any obvious movement of the air bubble (except a very small movement due to fluid filtration) during the course of the experiment indicated flow and served as a termination criterion for the experiment.

At the start of the experiment, the vessel was subjected to a pressure of 80 mmHg and brought back to its in-situ length, i.e. the length it had before it shortened during the isolation process. A temperature sensor and a pH electrode were placed into the experimental chamber. Heating of the experimental chamber was started until 37.0 ± 0.5 °C was achieved. The tolerated pH range was 7.4 ± 0.05. During the warm-up process, the vessel usually dilated almost completely and then contracted indicative of the development of a spontaneous myogenic tone serving as a first viability criterion (diameter less than 85% of the diameter of the initial dilation). After a stable diameter was established, smooth muscle contractility was further tested by application of 10^− 7^ M noradrenaline (viability criterion: at least 25% constriction), and endothelial function was checked by application of 10^− 6^ M acetylcholine (viability criterion: full dilation). Furthermore, the myogenic response was tested by exposing the vessel to static pressures of 40, 80, and 120 mmHg for 5 min each, pulsatile pressure with mean pressures of 40, 80, and 120 mmHg for 5 min each, and the initial static pressure series again. If stable responses to the pressure challenges were obtained, the specific experimental protocols were tested. At the end of each experiment, the maximum vessel diameter was determined using a calcium-free solution.

All data were expressed either as normalized diameter or myogenic tone. The normalized diameter is the actual diameter divided by the diameter measured in calcium-free solution at 80 mmHg to account for differences in vessel dimensions. Myogenic tone describes the momentary relative constriction of the vessel and is calculated as myogenic tone = 1 - dia_akt_/dia_max_ with dia_akt_ - actual diameter and dia_max_ - maximum diameter in calcium-free solution at the same pressure as dia_akt_.

### Calcium fluorimetry

Vessels were isolated, mounted, and tested for viability as described above. The fluorescent dye Fura-2 AM was used. Just before dye loading, background fluorescence of the mounted vessel was recorded. Loading was performed with 5 µM FURA-2 AM (dissolved in DMSO and diluted in physiological saline solution) for 60 min. The experimental chamber was then washed with physiological solution for 20 min. If the vessel still showed sufficient myogenic tone after this procedure, the experiment was started. FURA-2 was excited at 340 and 380 nm using a filter wheel and a xenon arc lamp. Emitted light was filtered at 520 nm with a long pass filter, collected by a photomultiplier, demultiplexed, and sent to a computer. There, the ratio of emission at the two excitation wavelengths (F340/F380) was calculated after subtraction of background fluorescence. Calibration of the ratio in terms of calcium was not performed because of the numerous uncertainties inherent to this method [13]. The changes in the FURA-2 ratio in response to pressure changes were normalized to the change in the FURA-2 ratio after addition of 60 mM KCl to account for differences in the reactivity of individual vessels.

### Drugs and chemicals

All salts for the solutions described above as well as acetylcholine and noradrenaline were from Sigma-Aldrich (Karlsruhe, Germany). FURA-2 AM was from Fisher Scientific (Schwerte, Germany). All chemicals and agents were stored based on the manufacturer’s recommendation. If necessary, a stock solution was prepared in the appropriate solvent, stored at −20 °C.

### Statistical analysis

All data are presented as mean ± standard error of the mean (SEM). Only one vessel was used per rat, so n corresponds to the number of rats, i.e. represents biological replicates. Statistical analysis was performed using SPSS 15.0. If a standard statistical test was not applicable due to different x-axis values of the curves to be compared, a suitable function (y = a*x + b or y = a*x^2^ + b*x + c) was fitted to the data and a statistical test of the function parameters was performed. Depending on the test design, the t-test or a repeated measures ANOVA was used. Statistical significance was accepted at *p* ≤ 0.05.

## Results

### The effect of pulsatile pressure is determined by its systolic pressure in rat small tail arteries

**Fig. 1:**
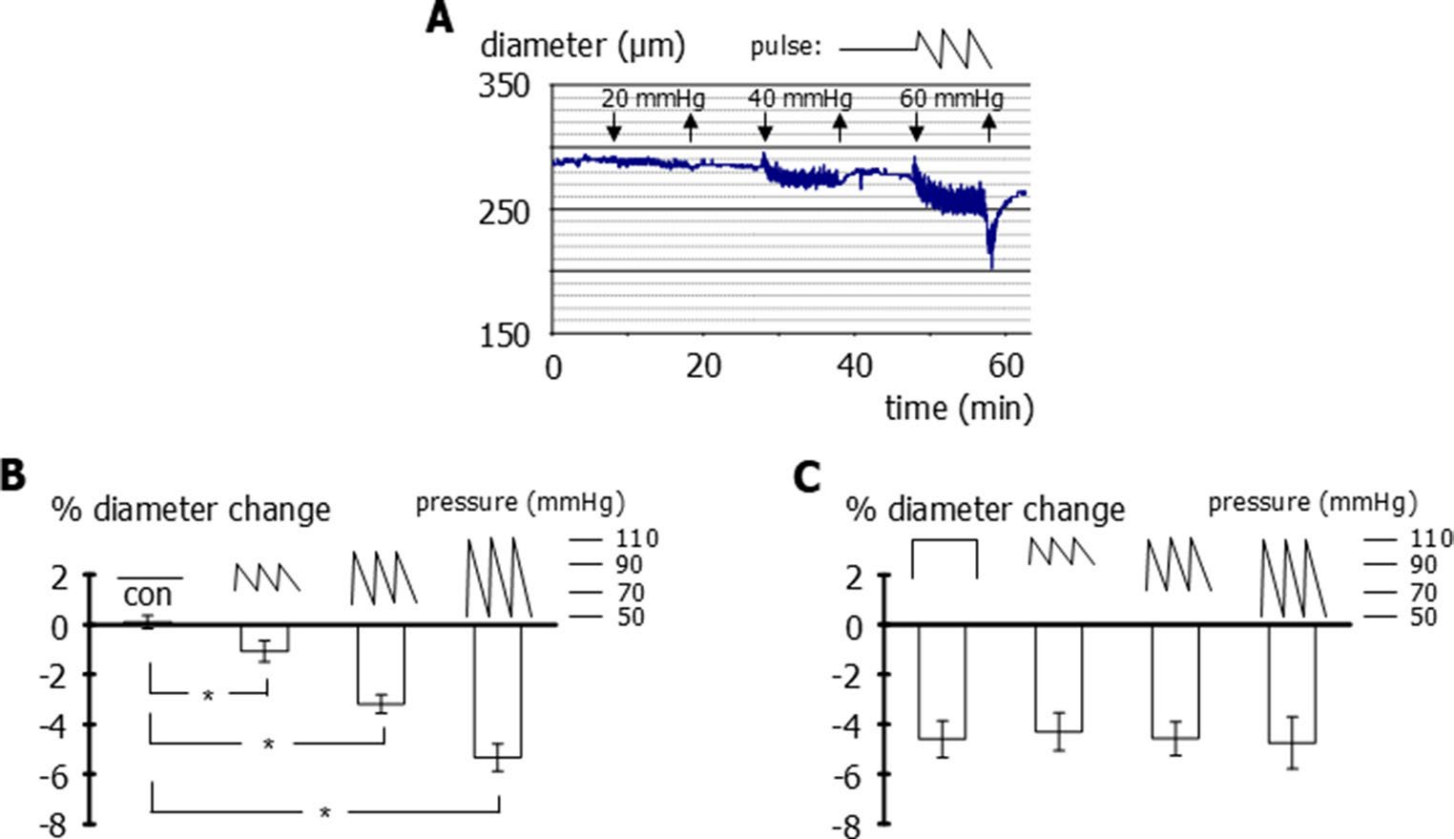
Effect of static and pulsatile pressure on the diameter of rat small tail arteries. **A**) Original recording of vessel diameter at a static pressure of 80 mmHg and in response to pulsatile pressure with different amplitudes (20, 40, and 60 mmHg) but unchanged mean pressure (80 mmHg). **B**) Diameter change (in % of initial diameter value) induced by static pressure at 80mmHg (con) and pulsatile pressure with different amplitudes (20, 40, and 60 mmHg) but unchanged mean pressure (80 mmHg). Repeated measures one-way ANOVA: *n*=6; p<0.001; multiple comparison with control (con) only by controlling for false discovery rate with two-stage linear step-up procedure of Benjamini, Krieger and Yekutieli: *n*=6; * - *p*=0.033, *p*<0.001, *p*<0.001, respectively. **C**) Diameter change (in % of initial diameter value) induced by a static pressure step (80 to 110 mmHg) and pulsatile pressure with different amplitudes (20, 40, and 60 mmHg) but unchanged systolic pressure (110 mmHg). Repeated measures one-way ANOVA: *n*=8; *p*=0.90

A first series of experiments was performed on rat small tail arteries, which possess a strong myogenic response [11, 14]. The pressure was altered from static to pulsatile with increasing amplitude (70 to 90, 60 to 100, and 50 to 110 mmHg), while maintaining a constant mean pressure of 80 mmHg. Pulsatile pressure induced vessel constriction, which became stronger with increasing amplitude (Fig. 1A, B). To explore whether this increasing constriction was evoked by the rising amplitude or the simultaneously rising systolic pressure, the latter was kept unchanged at 110 mmHg either during a static pressure step (80 to 110 mmHg) or during pulsatile pressure with increasing amplitude (90 to 110, 70 to 110, and 50 to 110 mmHg). We did not detect any difference between the various pressure modalities (Fig. 1C), suggesting that the systolic pressure level determines the effect of pulsatile pressure in rat small tail arteries.

**Fig. 2:**
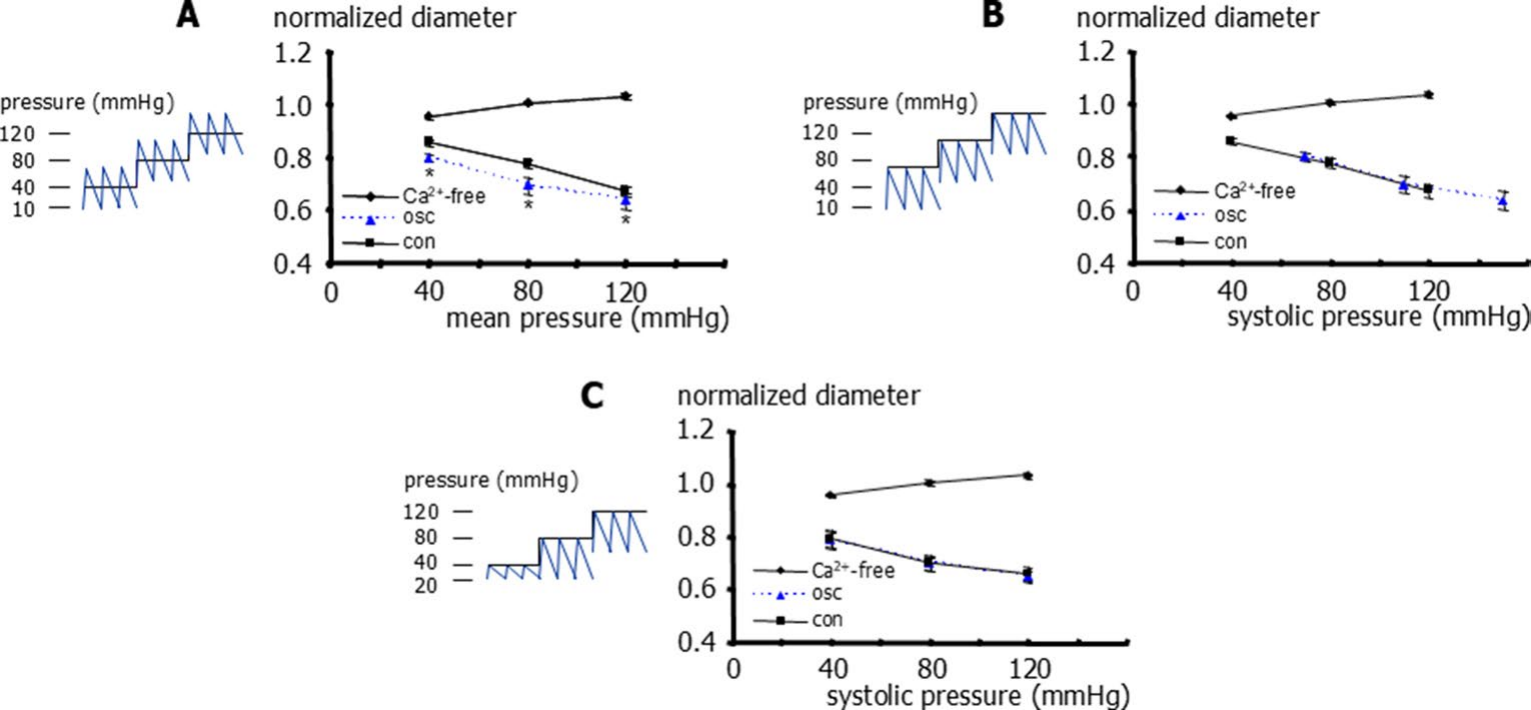
Effect of static and pulsatile pressure on the diameter of rat small tail arteries. **A**) Normalized vessel diameter at different levels of transmural pressure for static pressure (con), pulsatile pressure (10 to 70, 50 to 110, and 90 to 150 mmHg) expressed as its mean pressure (osc), and for static pressure in calcium-free experimental solution (Ca^2+^-free). Repeated measures two-way ANOVA (effect of treatment): *n*=5; *p*<0.001; multiple comparison by controlling for false discovery rate with two-stage linear step-up procedure of Benjamini, Krieger and Yekutieli (con vs osc): *n*=5; * - *p*=0.004 (40 mmHg), *p*<0.001 (80 mmHg),* p*=0.02 (120 mmHg), respectively. **B**) Normalized vessel diameter at different levels of transmural pressure for static pressure (con), pulsatile pressure (10 to 70, 50 to 110, and 90 to 150 mmHg) expressed as its systolic pressure (osc), and for static pressure in calcium-free experimental solution (Ca^2+^-free). Repeated measures two-way ANOVA for linear regression parameters (con vs osc): *n*=5; *p*=0.38. **C**) Normalized vessel diameter at different levels of transmural pressure for static pressure (con), pulsatile pressure (20 to 40, 20 to 80 and 60 to 120 mmHg) expressed as its systolic pressure (osc), and for static pressure in calcium-free experimental solution (Ca^2+^-free). Repeated measures two-way ANOVA (con vs osc): *n*=8; *p*=0.80

To further characterize the effect of pulsatile pressure on small tail arteries, a larger pressure range was explored. Static pressure steps of 40 to 80 to 120 mmHg were applied. The application of pulsatile pressure with mean pressures of 40, 80 and 120 mmHg and an amplitude of 60 mmHg (10 to 70, 50 to 110 and 90 to 150 mmHg) resulted in smaller diameters compared to static pressure conditions when the comparison was based on mean pressure (Fig. 2A). In contrast, when comparing these data based on systolic pressure, we did not detect any difference between static and pulsatile conditions (Fig. 2B). In this series, at a static pressure of 80 mmHg, the vessels had a diameter of 223 ± 7 μm (*n* = 5) in the experimental solution and 284 ± 5 μm (*n* = 5) in the calcium-free solution. Furthermore, we did not detect any difference between static (40, 80, and 120 mmHg) and pulsatile pressure with systolic pressures of 40, 80, and 120 mmHg (20 to 40, 20 to 80 and 60 to 120 mmHg) (Fig. 2C).

**Fig. 3:**
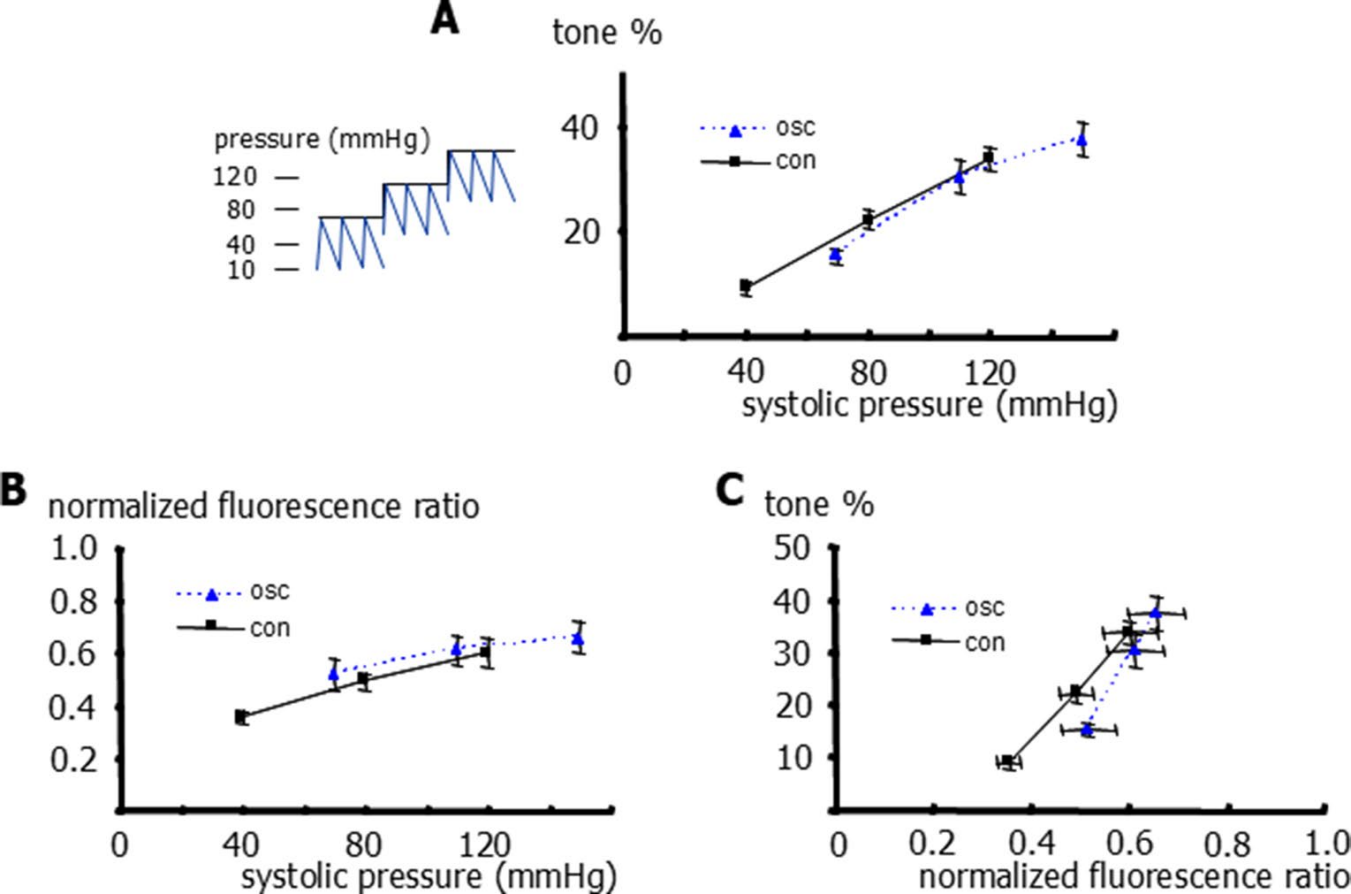
Effect of static and pulsatile pressure on tone, intracellular calcium and calcium sensitivity of rat small tail arteries.** A**) Vessel tone at different levels of transmural pressure for static pressure (con) and pulsatile pressure (10 to 70, 50 to 110, and 90 to 150 mmHg) expressed as its systolic pressure (osc). Repeated measures two-way ANOVA for linear regression parameters (con vs osc): *n*=5; *p*=0.90.** B**) Normalized fluorescence-ratio (F340/F380) at different levels of transmural pressure for static pressure (con) and pulsatile pressure (10 to 70, 50 to 110, and 90 to 150 mmHg) expressed as its systolic pressure (osc). Repeated measures two-way ANOVA for linear regression parameters (con vs osc): *n*=5; *p*=0.86. **C**) Relationship between the normalized fluorescence-ratio (F340/F380) and vessel tone for vessel responses induced by changes in static pressure (con) and pulsatile pressure (10 to 70, 50 to 110, and 90 to 150 mmHg) (osc). t-test for normalized fluorescence-ratio (F340/F380) at vessel tone of 25%: *n*=5; *p*=0.12

Furthermore, we did not detect any difference between vessel tone under conditions of static (40, 80 and 120 mmHg) and pulsatile pressure with an amplitude of 60 mmHg (10 to 70, 50 to 110 and 90 to 150 mmHg) when the comparison was based on systolic pressure (Fig. 3A). We also did not detect any difference between the intracellular calcium concentration under conditions of static and pulsatile pressure when the comparison was based on systolic pressure (Fig. 3B). From these data, the relationship between the intracellular calcium concentration and vessel tone, which represents the calcium sensitivity of myogenic tone, was derived. We did not detect any difference between the calcium sensitivity under conditions of static and pulsatile pressure; a myogenic tone of 25% was achieved at a normalized calcium of 0.51 ± 0.02 under static and of 0.58 ± 0.05 under pulsatile pressure (Fig. 3C).

### The effect of pulsatile pressure is determined by its mean pressure in rat gracilis arteries

**Fig. 4:**
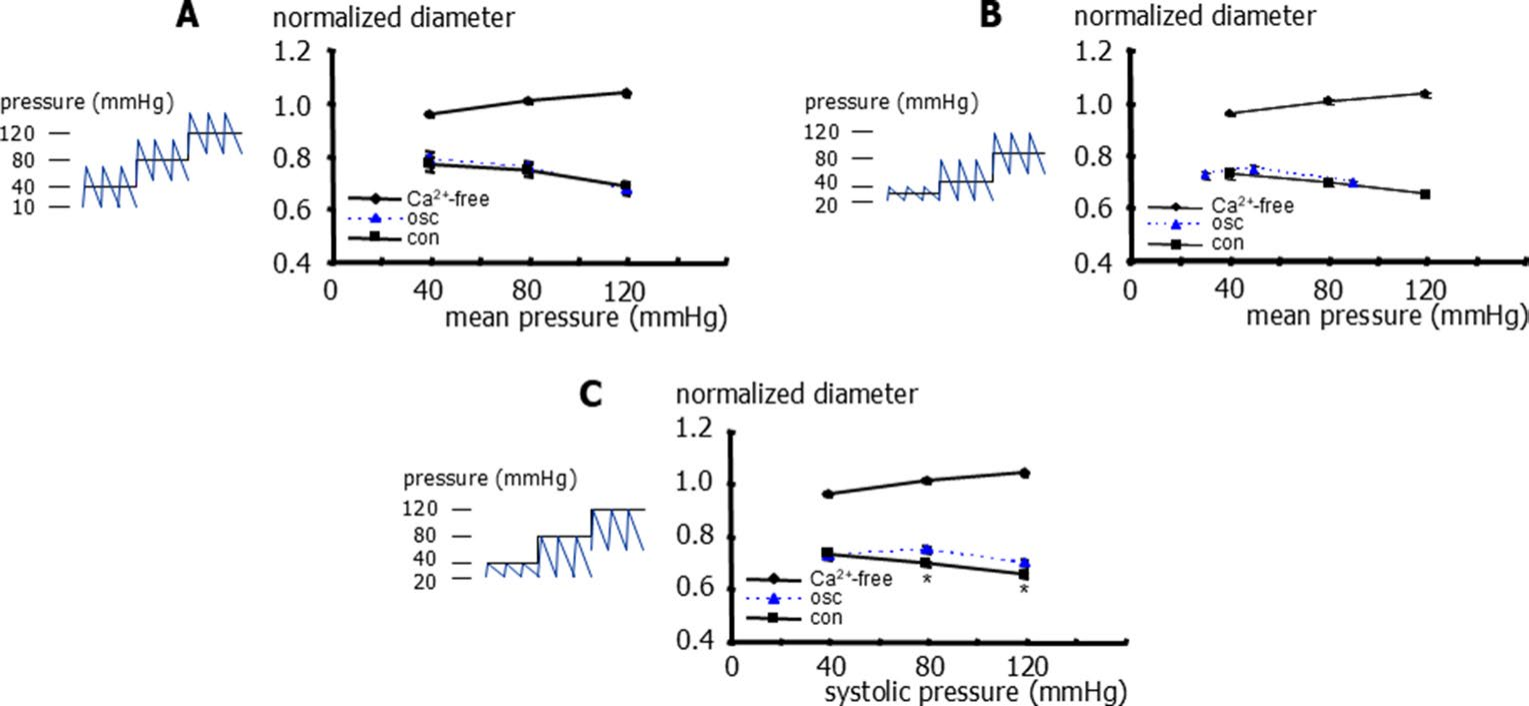
Effect of static and pulsatile pressure on the diameter of rat gracilis arteries. **A**) Normalized vessel diameter at different levels of transmural pressure for static pressure (con), pulsatile pressure (10 to 70, 50 to 110, and 90 to 150 mmHg) expressed as its mean pressure (osc), and for static pressure in calcium-free experimental solution (Ca^2+^-free). Repeated measures two-way ANOVA (con vs osc): *n*=6; *p*=0.54. **B**) Normalized vessel diameter at different levels of transmural pressure for static pressure (con), pulsatile pressure (20 to 40, 20 to 60 and 60 to 120 mmHg) expressed as its mean pressure (osc), and for static pressure in calcium-free experimental solution (Ca^2+^-free). Repeated measures two-way ANOVA for linear regression parameters (con vs osc): *n*=9; *p*=0.60. **C**) Normalized vessel diameter at different levels of transmural pressure for static pressure (con), pulsatile pressure (20 to 40, 20 to 80 and 60 to 120 mmHg) expressed as its systolic pressure (osc), and for static pressure in calcium-free experimental solution (Ca^2+^-free). Repeated measures two-way ANOVA (effect of pressure): *n*=9; *p*<0.001; multiple comparison by controlling for false discovery rate with two-stage linear step-up procedure of Benjamini, Krieger and Yekutieli (con vs osc): *n*=9; *p*=0.26 (40 mmHg), * - *p*=0.001 (80 mmHg), *p*=0.006 (120 mmHg), respectively

To further characterize the effect of pulsatile pressure on small arteries, another vessel, the gracilis artery, was examined. Static pressure steps of 40 to 80 to 120 mmHg were applied. When pulsatile pressure with mean pressures of 40, 80 and 120 mmHg and an amplitude of 60 mmHg (10 to 70, 50 to 110 and 90 to 150 mmHg) was applied, we did not observe any difference between static and pulsatile pressure when the comparison was based on mean pressure (Fig. 4A). In this series, at a static pressure of 80 mmHg, the vessels had a diameter of 209 ± 6 μm (*n* = 6) in the experimental solution and 279 ± 4 μm (*n* = 6) in the calcium-free solution. Furthermore, we did not detect any difference between static (40, 80 and 120 mmHg) and pulsatile pressure with systolic pressures of 40, 80 and 120 mmHg (20 to 40, 20 to 80 and 60 to 120 mmHg) when the comparison was based on mean pressure (Fig. 4B). In contrast, when the comparison of these data was based on systolic pressure, the application of pulsatile pressure resulted in larger diameters than under static pressure conditions (Fig. 4C).

**Fig. 5:**
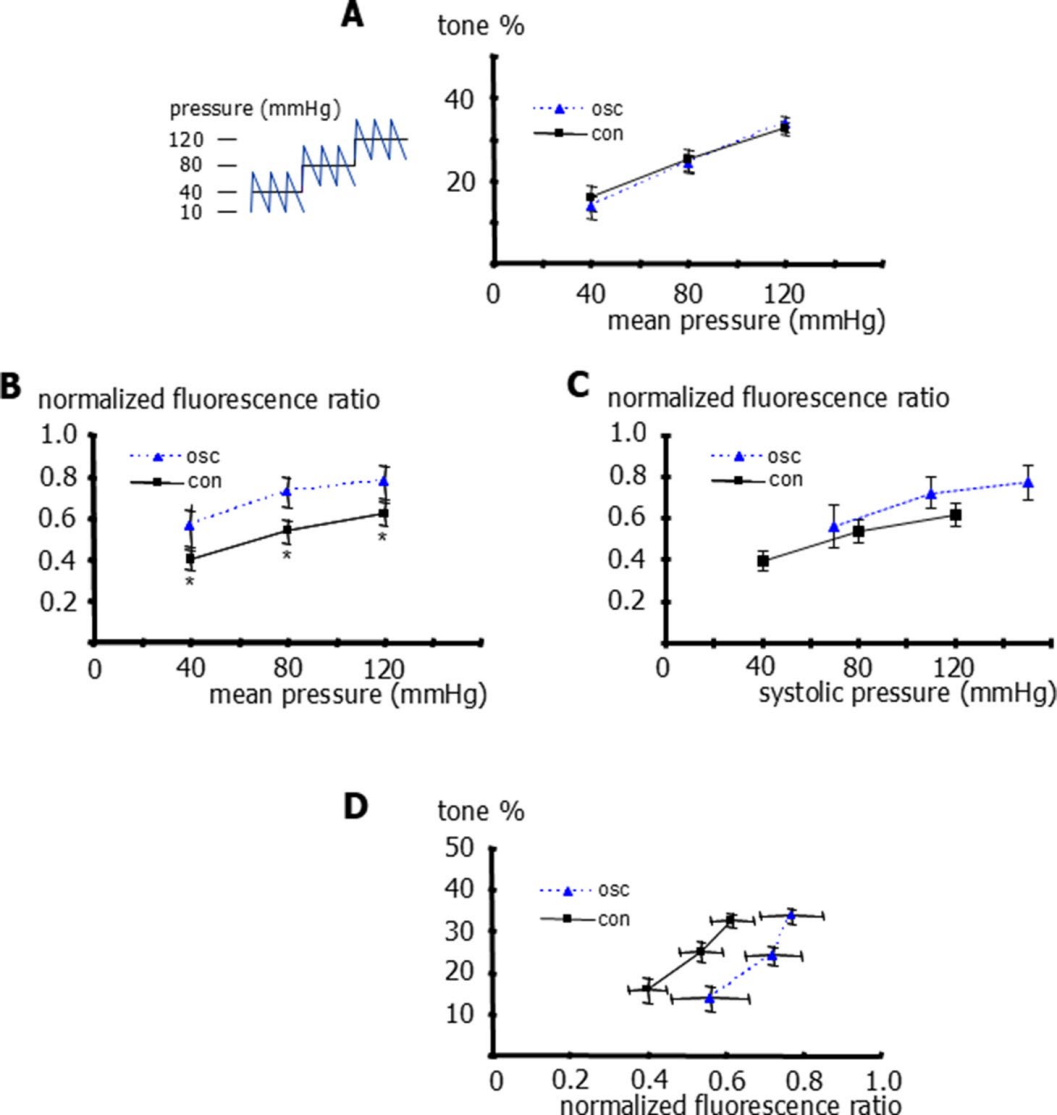
Effect of static and pulsatile pressure on tone, intracellular calcium and calcium sensitivity of rat gracilis arteries. **A**) Vessel tone at different levels of transmural pressure for static pressure (con) and pulsatile pressure (10 to 70, 50 to 110, and 90 to 150 mmHg) expressed as its mean pressure (osc). Repeated measures two-way ANOVA (con vs osc): *n*=6; *p*=0.51. **B**) Normalized fluorescence-ratio (F340/F380) at different levels of transmural pressure for static pressure (con) and pulsatile pressure (10 to 70, 50 to 110, and 90 to 150 mmHg) expressed as its mean pressure (osc). Repeated measures two-way ANOVA (effect of pressure): *n*=6;* p*<0.001; multiple comparison by controlling for false discovery rate with two-stage linear step-up procedure of Benjamini, Krieger and Yekutieli (con vs osc): *n*=6; * - *p*=0.02 (40 mmHg), *p*=0.009 (80 mmHg), *p*=0.01 (120 mmHg), respectively. **C**) Normalized fluorescence-ratio (F340/F380) at different levels of transmural pressure for static pressure (con) and pulsatile pressure (10 to 70, 50 to 110, and 90 to 150 mmHg) expressed as its systolic pressure (osc). Repeated measures two-way ANOVA for linear regression parameters (con vs osc): *n*=5; *p*=0.47 D) Relationship between the normalized fluorescence-ratio (F340/F380) and vessel tone for vessel responses induced by changes in static pressure (con) and in pulsatile pressure (10 to 70, 50 to 110, and 90 to 150 mmHg) (osc). t-test for normalized fluorescence-ratio (F340/F380) at vessel tone of 25%: *n*=5; *p*<0.05

Furthermore, we did not detect any difference between vessel tone under conditions of static (40, 80 and 120 mmHg) and pulsatile pressure with an amplitude of 60 mmHg (10 to 70, 50 to 110 and 90 to 150 mmHg) when the comparison was based on mean pressure (Fig. 5A). In contrast, the application of this pulsatile pressure resulted in higher intracellular calcium concentrations compared to static pressure conditions when the comparison was based on mean pressure (Fig. 5B). However, when the comparison was based on systolic pressure, we did not detect any difference between the intracellular calcium concentration under conditions of static and pulsatile pressure (Fig. 5C). From these data, the relationship between the intracellular calcium concentration and vessel tone, which represents the calcium sensitivity of myogenic tone, was derived. The application of pulsatile pressure resulted in weaker calcium sensitivity compared to static pressure conditions; a myogenic tone of 25% was achieved at a normalized calcium of 0.48 ± 0.09 under static pressure, but required a higher normalized calcium of 0.67 ± 0.09 under pulsatile pressure (Fig. 5D).

## Discussion

In this study, we explored the effect of pulsatile pressure on the myogenic response of rat small arteries. Our results show that pulsatile pressure affects the myogenic response in a vessel-specific manner. In small tail arteries, the effect of pulsatile pressure on the myogenic response is determined by its systolic pressure, whereas in gracilis arteries, the effect of pulsatile pressure is determined by its mean pressure. Interestingly, the effect of pulsatile pressure on the intracellular calcium concentration in both vessels is determined by its systolic pressure. However, while calcium sensitivity of myogenic tone did not differ between static and pulsatile pressure conditions in small tail arteries, calcium sensitivity in gracilis arteries was weaker under pulsatile pressure conditions than under static pressure conditions.

### Differential effect of pulsatile pressure on the myogenic response

In the present study, we observed that the effect of pulsatile pressure on the myogenic response in small tail arteries is determined by its systolic pressure and in gracilis arteries by its mean pressure.

These findings are consistent with previous reports, although only a few studies have examined differential effects of pulsatile pressure on the myogenic response. For example, pulsatile pressure induced endothelium-independent dilation of porcine coronary arterioles when static pressure of 60 mmHg was changed to pulsatile pressure with a mean value of 60 mmHg and an amplitude of 100 mmHg [7]. A similar response was observed in rat mesenteric arteries when static pressure of 50 mmHg was changed to pulsatile pressure with a mean pressure of 50 mmHg and an amplitude of 40 mmHg [15]. Such a dilating effect of pulsatile pressure was not observed in the present study. However, as in small tail arteries in the present study, pulsatile pressure evoked a constriction of rat afferent arterioles when static pressure of 100 mmHg was changed to pulsatile pressure with a mean value of 100 mmHg and an amplitude of 40 and 80 mmHg. Together with further experiments, the authors concluded that in this vessel type, the systolic pressure of the pressure pulse determines the myogenic response under pulsatile pressure conditions [8, 9]. Likewise, consistent with our results in gracilis arteries, vessel diameter in mouse middle cerebral arteries remained unchanged when static pressure at several levels was replaced with pulsatile pressure of the same mean value and a 40 mmHg amplitude [10].

Previous studies suggested that the organ-specific, differential effect of pulsatile pressure on the myogenic response serves organ-specific functional requirements. Thus, the dilatory effect of pulsatile pressure in coronary arterioles has been proposed to compensate for the compressive effects of cardiac contractions on these vessels [7]. In contrast, the contractile effect of pulsatile pressure, when the effect of pulsatile pressure is determined by its systolic pressure in afferent arterioles, has been proposed to protect the capillaries of the glomerulus from potentially damaging effects of systolic pressure. This was interpreted to demonstrate that renal protection is given priority over renal flow autoregulation [8, 9]. Furthermore, in cerebral arteries, the determination of the effect of pulsatile pressure by its mean value appears to give priority to blood flow autoregulation in this vascular bed [10]. Therefore, pulsatile pressure enables the determination of the myogenic response based on three factors, namely its systolic, mean, and diastolic pressure, allowing the myogenic response to be used more specifically for organ-specific regulation of the function of the circulatory system. However, to gain a more comprehensive understanding and enable a more generalized conclusion about the functional role of the differential effect of pulsatile pressure on the myogenic response, additional studies are needed that examine the effect of pulsatile pressure on the myogenic response from additional vascular beds and species.

Thus, our data reproduce and confirm the key findings of previous reports demonstrating an organ-specific, differential effect of pulsatile pressure on the myogenic response. In addition, our data extend previous findings by demonstrating differential effects of pulsatile pressure in two additional vessels, the small tail and the gracilis artery of the rat.

### Mechanism underlying the differential effect of pulsatile pressure on the myogenic response

The data from the present study show that in both vessels examined, the effect of pulsatile pressure on the intracellular calcium concentration was determined by its systolic pressure. To our knowledge, this is the first study addressing the effect of pulsatile pressure on the intracellular calcium concentration in the smooth muscle cells of the vessel wall. Thus, no direct comparison with previous results can be made. However, this new finding seems plausible when considering the well-established signaling pathways that control the myogenic response induced by static pressure (a summary of the evidence can be found in [1–5, 16–18]). In fact, one of the two major signaling pathways that control the myogenic response is a calcium-dependent pathway based on membrane potential depolarization and a subsequent increase of the intracellular calcium concentration, which is mainly due to influx of extracellular calcium, much of which is carried by voltage-operated calcium channels. Therefore, the differential effects of pulsatile pressure on the myogenic response of the two vessels studied cannot be explained by differences in intracellular calcium.

Interestingly, the data from the present study show that calcium sensitivity of myogenic tone in small tail arteries did not differ under static and pulsatile pressure conditions, whereas in gracilis arteries, calcium sensitivity was weaker under pulsatile than under static pressure conditions. Thus, more calcium must be made available in gracilis arteries under pulsatile pressure conditions to achieve the same relative vascular tone as under static pressure. This is the first study to examine the effect of pulsatile pressure on calcium sensitivity of myogenic tone. Thus, no direct comparison with previous results can be made. Nonetheless, this finding appears plausible in view of the well-established signaling pathways that control the myogenic response induced by static pressure [1–5, 16, 17]). In fact, the other signaling pathway that controls the myogenic response consists of a complex of several signaling mechanisms that regulate the calcium sensitivity of vessel tone, e.g., PKC, RhoA/Rho kinase/ROS (summarized evidence can be found in [1–6, 16–18]). The differential effect of pulsatile pressure on the myogenic response in the two vessels studied can therefore be explained by different calcium sensitization of myogenic tone under pulsatile pressure conditions.

The mechanisms of calcium sensitization of vessel tone in the myogenic response have so far been studied only under static pressure conditions. In particular, an earlier study on small tail arteries, one of the vessels explored in the present study, demonstrated that the Rho-kinase inhibitor Y27632 suppressed the myogenic response and produced a larger reduction of calcium sensitivity of vessel tone at 80mmHg than at 10mmHg [14]. Further evidence for the contribution of Rho-kinase, as well as PKC, to the myogenic response has also been obtained in mesenteric, cerebral, and skeletal muscle arteries [19–24]. Importantly, these findings showed that calcium sensitivity of myogenic tone consists of two successive elements: (i) the calcium sensitivity of the contractile apparatus, reflected by MYPT1-T855 and LC_20_ phosphorylation, and (ii) the “phosphorylation sensitivity” of myogenic tone, which describes how much myogenic tone is achieved at a given level of LC_20_ phosphorylation, that may be dependent on Rho-kinase- and PKC-mediated cytoskeletal reorganization [23, 25]. Notably, Rho-kinase probably plays a complex role in the myogenic response, affecting both the calcium-dependent pathway and the calcium-sensitivity pathway that control the myogenic response [5, 24, 26].

In summary, under static pressure conditions, the regulation of calcium sensitivity of the contractile apparatus during the myogenic response appears to be dominated by Rho-kinase. However, Rho-kinase also contributes to calcium-dependent signaling in the myogenic response. Rho-kinase, together with PKC, is involved in cytoskeletal reorganization in the myogenic response, i.e., in the regulation of the “phosphorylation sensitivity” of myogenic tone. Thus, myogenic tone depends in a complex manner on intracellular calcium, calcium sensitivity of the contractile apparatus, and the “phosphorylation sensitivity” of myogenic tone. Furthermore, Rho-kinase and PKC signaling are involved to varying degrees in several of these mechanisms. Therefore, delineating the mechanisms responsible for the organ-specific, differential effect of pulsatile pressure on the myogenic response requires a comprehensive investigation, which lies beyond the scope of the present study.

In conclusion, our data reproduce and confirm key findings of previous reports demonstrating an organ-specific, differential effect of pulsatile pressure on the myogenic response. Furthermore, our data extend previous findings by demonstrating differential effects of pulsatile pressure in two additional vessels, the small tail and the gracilis artery of the rat. Additional new findings show that a stronger myogenic response under pulsatile pressure conditions, i.e., the capability of a vessel to respond to systolic pressure, requires the vessel’s ability to maintain, and not lose, the calcium sensitivity of myogenic tone compared to static pressure conditions. Although our study focuses on mechanisms related to intracellular calcium (intracellular calcium concentration and calcium sensitivity) under pulsatile pressure conditions, it is limited in that it does not describe the following level of mechanisms, particularly those that control calcium sensitivity, as well as the implications of our findings for diseases associated with alterations in myogenic response. Since myogenic tone depends in a complex manner on several interacting signaling mechanisms involving Rho-kinase and PKC, delineating the mechanisms responsible for the organ-specific, differential effect of pulsatile pressure on the myogenic response, as well as their possible role in diseases, requires a comprehensive investigation in the future.

## Data Availability

The data that support the findings of this study are available in the Materials and Methods, Results, and/or Supplemental Material of this article.
